# Imatinib treatment reduces brain injury in a murine model of traumatic brain injury

**DOI:** 10.3389/fncel.2015.00385

**Published:** 2015-10-07

**Authors:** Enming J. Su, Linda Fredriksson, Mia Kanzawa, Shannon Moore, Erika Folestad, Tamara K. Stevenson, Ingrid Nilsson, Maithili Sashindranath, Gerald P. Schielke, Mark Warnock, Margaret Ragsdale, Kris Mann, Anna-Lisa E. Lawrence, Robert L. Medcalf, Ulf Eriksson, Geoffrey G. Murphy, Daniel A. Lawrence

**Affiliations:** ^1^Department of Internal Medicine, Division of Cardiovascular Medicine, University of Michigan Medical SchoolAnn Arbor, MI, USA; ^2^Department of Medical Biochemistry and Biophysics, Division of Vascular Biology, Karolinska InstitutetStockholm, Sweden; ^3^Molecular and Behavioral Neuroscience Institute, University of Michigan Medical SchoolAnn Arbor, MI, USA; ^4^Department of Molecular and Integrative Physiology, University of Michigan Medical SchoolAnn Arbor, MI, USA; ^5^Molecular Neurotrauma and Haemostasis, Australian Centre for Blood Diseases, Monash UniversityMelbourne, VIC, Australia

**Keywords:** traumatic brain injury, TBI outcome, blood brain barrier, platelet derived growth factor-CC, platelet derived growth factor receptor α, Imatinib, cerebral edema, Morris water maze

## Abstract

Current therapies for Traumatic brain injury (TBI) focus on stabilizing individuals and on preventing further damage from the secondary consequences of TBI. A major complication of TBI is cerebral edema, which can be caused by the loss of blood brain barrier (BBB) integrity. Recent studies in several CNS pathologies have shown that activation of latent platelet derived growth factor-CC (PDGF-CC) within the brain can promote BBB permeability through PDGF receptor α (PDGFRα) signaling, and that blocking this pathway improves outcomes. In this study we examine the efficacy for the treatment of TBI of an FDA approved antagonist of the PDGFRα, Imatinib. Using a murine model we show that Imatinib treatment, begun 45 min after TBI and given twice daily for 5 days, significantly reduces BBB dysfunction. This is associated with significantly reduced lesion size 24 h, 7 days, and 21 days after TBI, reduced cerebral edema, determined from apparent diffusion co-efficient (ADC) measurements, and with the preservation of cognitive function. Finally, analysis of cerebrospinal fluid (CSF) from human TBI patients suggests a possible correlation between high PDGF-CC levels and increased injury severity. Thus, our data suggests a novel strategy for the treatment of TBI with an existing FDA approved antagonist of the PDGFRα.

## Introduction

The Centers for Disease Control estimate that every year in the United States approximately 2.5 million people sustain a Traumatic brain injury (TBI). There are approximately 53,000 TBI related deaths and 283,000 hospitalizations annually, with many patients suffering permanent disability (Frieden et al., 2014). Additionally, TBI is a contributing factor in nearly a third of all injury-related deaths in the United States and is a leading cause of death in North America for individuals between the ages of 1–45 (Rutland-Brown et al., 2006; Hemphill and Phan, 2013b; Byrnes et al., 2014). TBI also accounts for more lost work time than cancer and cardiovascular diseases combined (Thurman et al., 1999; Ma et al., 2014). Over the past two decades our understanding of the complex pathobiology of TBI has improved significantly. However, despite numerous studies in animal models of TBI and clinical trials of various therapeutic strategies, no effective therapy for TBI patients has emerged (Grumme et al., 1995; Marmarou et al., 1999; Bramlett and Dietrich, 2004; Yurkewicz et al., 2005; Maas et al., 2006, 2010). The pathophysiology of TBI is complex and involves both primary and secondary insults (Hemphill and Phan, 2013b; Finnie, 2014). Primary injury to the brain can be induced by numerous mechanisms, such as brain contusion, hematoma, and shearing or stretching of the brain tissue caused by motion of the brain structures relative to the skull. Secondary injury development includes multifaceted biochemical and physiological processes that are initiated by the primary insult and manifest over a period of hours to days and even months (Cernak, 2005; Finnie, 2014; Logsdon et al., 2015).

The lack of effective pharmacological treatments for TBI patients despite the many clinical trials in the past two decades suggests that the development of improved therapies for the treatment of TBI will depend upon a better understanding of the underlying mechanisms that drive secondary neuronal injury during the acute phase of TBI. One of the most serious and difficult to control secondary effects of TBI is the development of cerebral edema. Cerebral edema leads to brain swelling and increased intracranial pressure (ICP), which in severe cases can result in cistern compression, brain herniation, and even death. The causes of edema in TBI patients are complex but it is well appreciated that the loss of the blood brain barrier (BBB) is a significant factor in the development of vasogenic edema (Chodobski et al., 2011). Our recent studies, and those of others, have shown that signaling through the PDGF receptor α (PDGFRα) in the neurovascular unit (NVU) can promote BBB permeability and neuronal injury in several different neuropathological settings, including ischemic and hemorrhagic stroke, spinal cord injury, MS, and seizures (Su et al., 2008; Ma et al., 2011; Abrams et al., 2012; Adzemovic et al., 2013; Fredriksson et al., 2015). In our previous studies of ischemic stroke we have found that the protease tissue-type plasminogen activator (tPA) induces opening of the BBB through proteolysis of latent platelet derived growth factor-CC (PDGF-CC), generating an active form of PDGF-CC that binds to the PDGFRα and induces cell signaling (Su et al., 2008). The PDGFRα is localized to astrocytes in the NVU (Su et al., 2008; Fredriksson et al., 2015), and blocking this pathway with either the PDGFRα antagonist Imatinib or neutralizing antibodies to PDGF-CC reduces BBB dysfunction and improves outcome after ischemic stroke (Su et al., 2008). Similar results have been obtained by blocking PDGFRα signaling in animal models of hemorrhagic stroke, spinal cord injury, MS, and seizures (Ma et al., 2011; Abrams et al., 2012; Adzemovic et al., 2013; Fredriksson et al., 2015). These latter studies suggest that blocking PDGFRα signaling may provide benefit in diverse CNS pathologies through protection of the BBB. Consistent with this suggestion our recent work indicates that tPA can promote post-traumatic cerebrovascular damage including increased BBB leakage (Sashindranath et al., 2012). However, it is not known whether the PDGFRα pathway also plays a role in TBI-related injuries.

In the study presented here we used two versions of a well-established mouse model of TBI, controlled cortical impact (CCI; Sinz et al., 1999; Gilmer et al., 2008; Loane et al., 2009) to demonstrate for the first time that Imatinib treatment after TBI reduces BBB opening and significantly improves outcomes. Our data suggest that PDGF signaling contributes to the development of vasogenic edema by increasing BBB opening after TBI and that both vasogenic edema and cognitive impairment can be reduced by Imatinib treatment. These findings identify novel targets for TBI treatment and contribute to our understanding of the relationship between BBB leakage and the downstream secondary injuries associated TBI. In addition, we demonstrate the potential effectiveness of Imatinib, an existing FDA approved inhibitor of the PDGFRα pathway, for the treatment of acute TBI, suggesting the possibility of rapid translation of these results.

## Material and Methods

### TBI Models

Ten-week-old male C57BL/6J mice were anesthetized with 2% isoflurane and placed in a stereotatic frame (Kopf, Tujunga, CA, USA). Core body temperatures were maintained at 37.0° C for the entire procedure. For the unilateral TBI experiments, a 3.5 mm craniotomy was made over the right parietotemporal cortex with an electric drill (Harvard Apparatus) and the bone flap was removed. Vertically directed CCI was performed using a pneumatic impactor (Precision Systems and Instrumentation, VA) with a 3 mm flat-tip. The impact speed, tissue displacement and impact duration were set at 3.65m/s, 1 mm, and 400 ms respectively. A cap made from Dental Acrylic was glued to cover the craniotomy. To generate a larger bilateral injury, a previously described TBI model was used where the CCI is delivered to the midline (Liu et al., 2013). For this model a 5 mm circular craniotomy was made with center near bregma −2.5 and the impact speed, tissue displacement and impact duration were set at 3.00m/s, 1.1 mm, and 50 ms respectively. After the impact, the circular bone fragment from the craniotomy was glued back to the cranial window. For the unilateral TBI experiments, animal group sizes were *n* = 10 for the Evans blue (EB) Assays, *n* = 6 in the T2 and apparent diffusion co-efficient (ADC) analysis, and *n* = 5 for volumetric tissue loss after 21 days. In the bilateral TBI experiments, animal group sizes were *n* = 5 in the T2 and ADC analysis, *n* = 5 for volumetric tissue loss after 21 days, and *n* = 7–9 in the Morris water maze (MWM) studies. Separate groups of animals were used in each experiment and were not overlapped with the exception that the T2 and ADC analysis at 24 h and 7 days were performed on the same mice. For sham surgeries, all animals underwent the same surgical procedures except the craniotomy and CCI. All animals received the analgesic carprofen (5 mg/kg by subcutaneous injection) immediately prior to surgery, and post-surgery care consistent with the “*Guide for the Care and Use of Laboratory Animals*”. Briefly, mice were kept on a 37°C warming pad overnight during recovery and were monitored daily for any distress behavior until the end of the study, receiving analgesics after surgery as needed. All animal experiments were approved by the University Committee on Use and Care of Animals at the University of Michigan, and conducted in accordance with the United States Public Health Service’s Policy on Humane Care and Use of Laboratory Animals. In general, the mice tolerated the procedures well. They were lethargic in the first few hours after surgery, and mice with the bilateral injury took longer to recover from anesthesia than mice in the unilateral model and remained lethargic for a longer period of time during the first day. There were no deaths or other complications with the unilateral model, but there was 1 death out of 52 mice subjected to the bilateral injury.

### Evans Blue Analysis

For analysis of cerebrovascular permeability after TBI, mice were injected with 100 microliters of 4% EB (intravenous, Sigma-Aldrich) in lactated Ringer’s solution 1 h before the animals were sacrificed by transcardial perfusion with phosphate buffered saline (PBS) for 8 min. The brains were removed and separated into hemispheres ipsilateral and contralateral to the TBI. Each hemisphere was then homogenized in N, N-dimethylformamide (Sigma-Aldrich) and centrifuged for 45 min at 25,000 rcf. The supernatants were collected and quantitation of EB extravasation performed as described (Yepes et al., 2003). Briefly, EB levels in each hemisphere were determined from the formula:

$$\left({\text{A}}_{\text{620nm}}-\left(\left({\text{A}}_{\text{500nm}}{\text{+ A}}_{\text{740nm}}\right)/\text{2}\right)\right)/\text{mg}\text{ wet weight.}$$

### MRI Scan

After CCI, animals were anesthetized with 2% isoflurane/air mixture for T2 scans (7.0T Varian MR, 183 mm horizontal bore, Varian, Palo Alto, CA, USA). A double-tuned volume radiofrequency coil was used to scan the head region of the mice. Axial T2-weighted images were acquired using a fast spin-echo sequence with the following parameters: repetition time (TR)/effective echo time (TE), 4000/60 ms; echo spacing, 15 ms; number of echoes, 8; field of view (FOV), 20 × 20 mm; matrix, 256 × 128; slice thickness, 0.5 mm; number of slices, 25; and number of scans, 1. The protocol for diffusion weighted imaging (DWI) utilized the following parameters: TR/TE, 4000/47 ms; FOV, 20 × 20 mm; matrix, 128 × 64 and the same slice package as the above spin-echo sequence.

For the data analysis, Image J software (NIH) was used to calculate the lesion volume from T2 scan, and Matlab software (MathWorks, Natick, MA, USA) was used to calculate the apparent diffusion coefficient from DWI scan. To calculate volumetric tissue loss 21 days after TBI, ROIs from MRI slices corresponding to the hippocampal region were calculated by Image J. The volume scales used (mm^3^) for all T2 scans were the same in each model.

### Imatinib Treatment

To block PDGFRα activation, mice were treated twice daily by oral gavage with the tyrosine kinase inhibitor Imatinib (200 mg/kg) starting 45 min after TBI and repeated for 5 days. Lactated Ringer’s solution was used as vehicle control.

### Histology

Deeply anaesthetized mice were perfused transcardially 21 days after TBI with PBS for 2 min and followed by 4% paraformaldehyde for 5 min. After perfusion, the brains were quickly removed from the skull and post-fixed for 1 h in buffered 4% paraformaldehyde (+4 °C), and embedded in OCT and stored at –70 °C until cut. Brains were cut in 14 μm-thick coronal sections with a sliding microtome and stained with hematoxylin and eosin. Images were visualized using Nikon Eclipse TE-2000e and captured with a digital camera (Q-Imaging Micropubliser RTV version 5.0). These captured images from each section were then stitched together by metamorph software (version 7.7.4.0).

### Morris Water Maze

The MWM was performed as previously described (McKinney et al., 2008; White et al., 2008; Perkowski and Murphy, 2011). The pool was 1.2 meters in diameter and filled with water made opaque with white non-toxic paint. The escape platform consisted of a 10 cm platform that was submerged 0.5 cm below the surface of the water in the center of one of the quadrants. Water was maintained at 25 ± 2° C. The walls surrounding the pool were adorned with high-contrast posters for use as distal cues. The room was lit by indirect white light (200 lux in center of pool).

For 10 days prior to training, mice were handled for 2–3 min once daily. Every training trial began with the mouse on the platform for 15 s. The mouse was then placed into the water facing the wall of the pool and allowed to search for the platform. The trial ended either when the mouse climbed onto the platform or when 60 s had elapsed. At the end of each trial the mouse was allowed to rest on the platform for 15 s. Mice were given 6 trials per day (in blocks of two trials, 1 min inter-trial intervals and 1 h inter-block intervals) for 4 days, with the starting position chosen pseudo-randomly among 6 start positions. A probe trial was conducted 24 h after the end of training (on day 5). During the probe trial, the escape platform was removed and mice were placed in the pool at the start location directly opposite of where the platform was previously located and allowed to swim for 60 s. To control for motivation and sensory deficits mice were also examined in the visible-platform version of the MWM 24 h after the probe trial. The visible-platform version consisted of a single day of training with 6 trials during which the platform was moved to a new location and marked with a distinct local cue. All MWM data was acquired with a digital video camera 1.5 meters from the water surface. Images from the digital camera were processed and stored on a desktop PC using Actimetrics WaterMaze Software (Actimetrics, Wilmette, IL). Water maze performance was quantified using proximity. Proximity measurements were calculated by the tracking software at a rate of 1 Hz as the instantaneous distance (in centimeters) from the designated platform location. We prefer to use proximity measures because they are independent of swim speed (Gallagher et al., 1993) and moreover, the average proximity measure is the most sensitive probe trial measurement (Maei et al., 2009). Performance during training was assessed using a cumulative proximity measure. Cumulative proximity is the sum of all of the instantaneous distance measurements minus the distance calculated as a perfect swim path which is represented as a straight line between the start location and the platform location. Performance during the probe trial was assessed using an average proximity measure. Average proximity is the average of all of the instantaneous distance measurements recorded during the probe trial with lower values reflecting a more selective search strategy.

### Human CSF Samples

This study was conducted in accordance with the National Health and Medical Research Council of Australia National Statement on Ethical Conduct in Research Involving Humans and approved by Human Ethics Committees of the Alfred Hospital and Austin Hospital, Melbourne, Australia. Cerebrospinal fluid (CSF) was obtained from 17 TBI patients (16 male; 1 female) aged between 15 and 58 years (mean 31.1 years) recruited via the Trauma Service of the Alfred Hospital with delayed informed consent. Patients who required an external ventricular drain (EVD) for monitoring ICP were included. The CSF was collected within the first 36 h of admission in bags over 24 h in cooled containers then centrifuged at 2000 g for 15 min at 4°C and stored at –80°C. Non-TBI control CSF samples obtained from 4 patients recruited to the Alfred hospital for elective neurosurgery, or via lumbar puncture from 5 patients recruited to the Austin Hospital (Melbourne) suspected of having multiple sclerosis. CSF samples were centrifuged as above and the supernatants stored at −80°C.

Western blot analysis was performed to detect PDGF-CC levels in human CSF samples. CSF (30 μl of undiluted CSF) were subjected to SDS-PAGE electrophoresis under denaturing conditions and transferred to nylon membranes using standard conditions. To detect PDGF-CC, membranes were hybridized with a polyclonal goat anti-human PDGF-C antibody (RnD Systems, AF1560) diluted 1:200. After washing, membranes were hybridized with HRP tagged secondary rabbit anti-goat IgG antibody (1:5000 dilution). PDGF-CC signals were revealed using enhanced chemiluminescence (SuperSignal West Pico; Thermo Scientific, Rockford, IL, USA). The relative amount of PDGF-CC in each sample was estimated from the intensity of the PDGF-CC band in each lane and assigned a value from 0 to 10, with 0 being undetected and 10 being the most intense. The average for all the samples at each extended glasgow outcome scale (GOSE) score was plotted against the GOSE score.

### Statistical Analysis

The data are presented as mean ± SEM. All statistical analysis was performed using GraphPad Prism statistical software. Significant outliers were identified and excluded based on Grubb’s test with significance level alpha set to 0.05. Analysis for significance was performed as indicated in the figure legends. All neuropathology experiments were repeated at least two independent times and *n* indicates the number of individual mice used in the study. *P* values less than 0.05 were considered statistically significant and are indicated in the figures by an asterisk.

## Results

### Imatinib Inhibits the BBB Leakage after TBI

**Figure 1:**
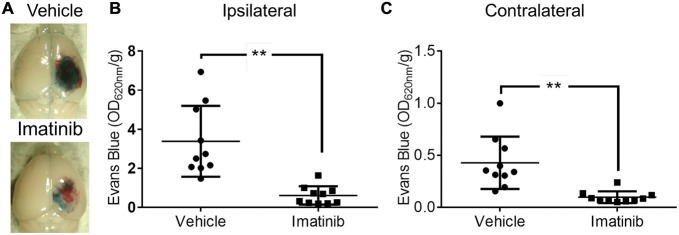
**Imatinib reduces blood brain barrier (BBB) leakage after Traumatic brain injury (TBI).** Mice were treated with vehicle or Imatinib (200 mg/kg, daily p.o. for 1 day) starting 45 min after unilateral controlled cortical impact (CCI). Twenty three hours after unilateral CCI, Evans blue (EB) dye was injected intravenously as a bolus and animals were perfused with phosphate buffered saline (PBS) 1 h later. **(A)** Representative brains from vehicle and Imatinib treated animals are shown. **(B,C)** Quantitative analysis of EB leakage in the ipsilateral **(B)** and contralateral **(C)** hemispheres. Data are expressed as mean ± SEM (*n* = 10) and double asterisks indicate *p* < 0.01.

Since both TBI and ischemic stroke share the common pathophysiologic mechanisms of loss of BBB control and the development of cerebral edema (Bouma et al., 1991; Marion et al., 1991; Zauner et al., 1996; Bramlett and Dietrich, 2004; Lescot et al., 2010), we hypothesized that, as is the case with ischemic stroke, PDGFRα signaling might also play a role in TBI. To test this hypothesis we investigated if blocking PDGFRα signaling after TBI could reduce BBB leakage. For these studies we examined the efficacy of treatment with the PDGFRα antagonist Imatinib in mice subjected to CCI in the right parietotemporal cortex. The mice were treated with either vehicle or 200 mg/kg of Imatinib by gavage 45 min after CCI and again 22 h after injury. The mice were then analyzed for BBB integrity by EB extravasation 24 h after TBI. We predicted that if PDGFRα signaling contributes to loss of BBB integrity after TBI then Imatinib treatment should result in less BBB leakage 24 h after TBI. Consistent with our hypothesis, we found that compared to vehicle-treated controls, Imatinib treatment significantly reduced EB leakage in the ipsilateral hemisphere 24 h after TBI (Figures 1A,B >70%). We also found that the injury resulted in BBB leakage in the contralateral hemisphere, but at levels that were nearly 8-fold lower than in the ipsilateral hemisphere (3.4 ± 0.6 ipsi vs. 0.43 ± 0.08 contra). Similar to the effects observed in the ipsilateral hemisphere Imatinib treatment was also effective at reducing BBB leakage in the contralateral hemisphere (Figure 1C >75%). These results demonstrate that like its effects in stroke, spinal cord injury and MS (Su et al., 2008; Ma et al., 2011; Abrams et al., 2012; Adzemovic et al., 2013), Imatinib treatment preserves BBB integrity following TBI.

### Imatinib Reduces Lesion Size and Vasogenic Edema after TBI

**Figure 2:**
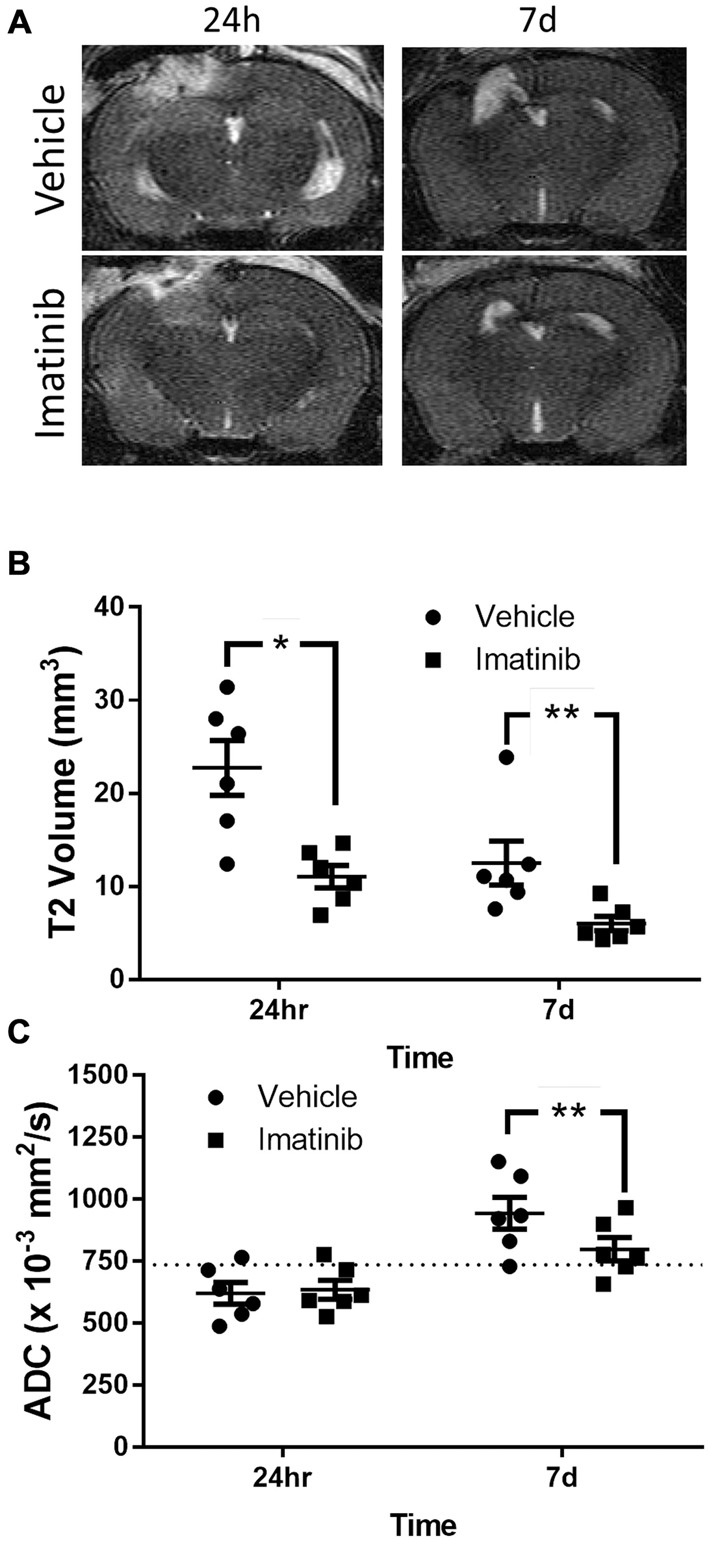
**Imatinib reduces lesion volume and edema after TBI.** Mice were treated with vehicle or Imatinib (200 mg/kg, twice daily p.o. for 5 days) starting 45 min after unilateral CCI and examined for lesion volume and cerebral edema by MRI 24 h and 7 days after unilateral CCI. **(A)** Representative T2-weighted MRI images. **(B)** Quantitative analysis of the lesion volume determined from theT2-weighted hyper-intense signal. **(C)** The relative extent of cerebral edema in the ipsilateral hemisphere was determined by calculating the apparent diffusion co-efficient (ADC) values from diffusion weighted imaging (DWI) scans. Data are expressed as mean ± SEM (*n* = 6); single asterisk indicates *p* < 0.05, double asterisks indicate *p* < 0.01, and the dotted line indicates the normal ADC value of 736 × 10^−3^ mm^2^/s reported for age matched mice (Rau et al., 2006).

Since brain edema is a common and serious consequence of TBI that contributes to lesion expansion and secondary brain damage (Dietrich et al., 1994; del Zoppo and Mabuchi, 2003) and since the rise in edema after TBI is thought to be due, at least in part, to the loss of the BBB control, we examined if Imatinib treatment also reduced lesion size and edema 24 h and 7 days after TBI. For these studies mice were subjected to CCI in the right parietotemporal cortex and then treated with either vehicle or 200 mg/kg of Imatinib by gavage 45 min after TBI and then twice a day for 5 days following injury. T2 and DWI MRI scans were obtained at 24 h and 7 days post-injury. The T2 scans were used to calculate the lesion volume and showed that by 24 h after CCI there was conspicuous tissue damage within the region surrounding the impact site (Figure 2A). The T2 scans also revealed that by 7 days post-injury there was extensive tissue loss and cavitation in the cortex of untreated mice that was significantly reduced in the Imatinib treated group (Figure 2A). Quantification of these data demonstrated that at 24 h after injury the mean T2 lesion volume was 22.7 ± 2.9 mm^3^ in the vehicle-treated group and 11.1 ± 1.2 mm^3^ in the Imatinib-treated group. By 7 days after injury, the T2 signal was reduced in both groups. However, the Imatinib-treated mice still showed a significant reduction in lesion size of approximately 50% compared to the vehicle-treated group (12.5 ± 2.4 mm^3^ vs. 6.0 ± 0.8 mm^3^, Figure 2B).

To examine the effect of Imatinib treatment on edema in the injured hemisphere, the DWI scans of these mice were used to calculate hemispheric ADC values, which measure the impedance of water molecule diffusion within tissue. These results are shown in Figure 2C. In both groups the ADC values were reduced by 24 h after TBI compared to uninjured mice (Rau et al., 2006) (the normal ADC value is shown by the dashed line in Figure 2C). This indicates the presence of cytotoxic edema in both vehicle- and Imatinib-treated mice that was not significantly different between the groups. By day 7 there was a pseudo-correction of the ADC above the normal value indicating the development of vasogenic edema in the vehicle-treated mice. However, in the Imatinib-treated group the ADC value was significantly lower, indicating that there was much less vasogenic edema 7 days after injury in Imatinib treated mice (0.944 ± 0.065 mm^2^/s vs. 0.799 ± 0.046 mm^2^/s, Figure 2C).

### Imatinib Reduces Cavitation and Tissue Loss after TBI

**Figure 3:**
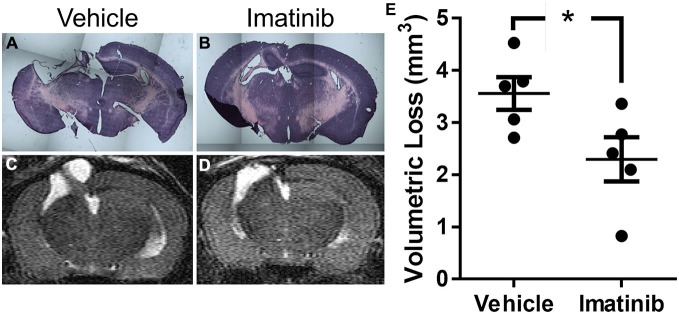
**Imatinib reduces tissue loss after TBI.** Mice were treated with vehicle or Imatinib (200 mg/kg, twice daily p.o. for 5 days) starting 45 min after unilateral CCI. Twenty one days later, animals underwent MRI T2 scan and then were perfused and fixed in paraformaldehyde and stained with H&E. **(A,B)** Representative photomicrographs of coronal sections near bregma −1.5 (10× objective) from vehicle-treated **(A)** and Imatinib-treated **(B)** mice. **(C,D)** Representative T2-weighted MRI images from vehicle-treated **(C)** and Imatinib-treated mice **(D). (E)** Quantification of volumetric loss of tissue determined from theT2-weighted hyper-intense signal. Data are expressed as mean ± SEM (*n* = 5) and the single asterisk indicates *p* < 0.05.

Since neuronal loss in the hippocampus has been linked to pathological outcomes of TBI in both humans (Tate and Bigler, 2000; Swartz et al., 2006) and in animal models of TBI (McIntosh et al., 1989; Dixon et al., 1991; Lowenstein et al., 1992; Hall et al., 2008), we next investigated whether intervention in the acute phase of TBI with 5 days of Imatinib treatment translated into long term tissue preservation. For these studies mice were subjected to CCI in the right parietotemporal cortex and then treated with either vehicle or 200 mg/kg of Imatinib by gavage 45 min after CCI and then twice a day for 5 days following injury. Previous studies have demonstrated that hippocampal neurons undergo regional alterations and remodeling after TBI (Witgen et al., 2005; Cohen et al., 2007). Consistent with this, hematoxylin and eosin staining of brain sections showed that there was dramatic tissue loss and remodeling in the cortex and hippocampus 21 days after TBI, including large cavitations (Figure 3A). As with BBB permeability, lesion size, and vasogenic edema, Imatinib substantially reduced cavitations (Figure 3B). To quantify the extent of the cavitations, MRI T2 scans were performed 21 days after TBI. These scans confirmed the loss of tissue noted in the H&E stains, showing large areas of hyper-intense signals indicative of cavitation (Figures 3C,D). Quantification of the hyper-intense signals showed that Imatinib treatment significantly reduced tissue loss (vehicle 3.6 ± 0.3 mm^3^ vs, Imatinib 2.3 ± 0.4 mm^3^ Figure 3E). This suggests that early intervention with Imatinib may impact long term TBI progression.

### Imatinib Protects in a Bilateral TBI Model

Since neuronal loss and remodeling can affect long term memory and cognitive function, mice that were subjected to an identical injury and treatment were subsequently examined in the MWM test. However, neither the vehicle- nor the Imatinib-treated mice showed a consistent deficit, suggesting that the injury to one hemisphere may not be extensive enough to result in measurable behavioral changes (not shown). Consistent with this interpretation several studies have suggested that when an undamaged unilateral hippocampus is present, behavioral changes in mice are minimized in memory and cognition tests (Shipton et al., 2014). Based on these recent studies, we hypothesize that the lack of significance in our MWM test is due to the presence of an intact hippocampus on the contralateral side resulting in the lack of a measureable cognitive deficit in the control cohort. We therefore extended our study with a second, more severe, TBI model where CCI is delivered to the midline producing a larger bilateral TBI (Liu et al., 2013).

**Figure 4:**
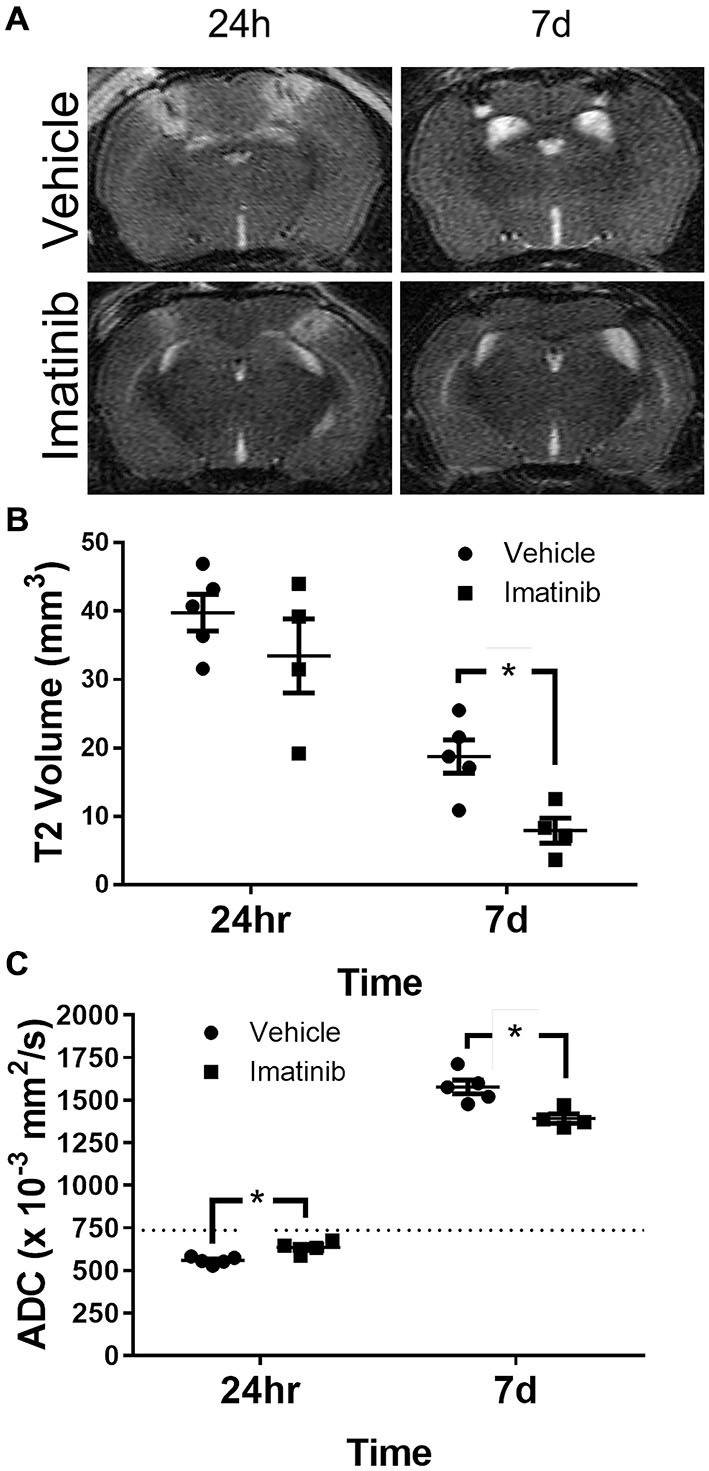
**Imatinib reduces lesion volume and edema after Bi-lateral TBI.** Mice were treated with vehicle or Imatinib (200 mg/kg, twice daily p.o. for 5 days) starting 45 min after bilateral CCI and examined for lesion volume and cerebral edema by MRI 24 h and 7 days after bilateral CCI. **(A)** Representative T2-weighted MRI images. **(B)** Quantitative analysis of the lesion volume determined from theT2-weighted hyper-intense signal. **(C)** The relative extent of cerebral edema in both hemispheres was determined by calculating the ADC values from DWI scans. Data are expressed as mean ± SEM (*n* = 4–5), the single asterisk indicates *p* < 0.05, and the dotted line indicates the normal ADC value of 736 × 10^−3^ mm^2^/s reported for age matched mice (Rau et al., 2006).

To characterize the extent of injury in the midline CCI model and to examine the potential efficacy of Imatinib in a more severe model of TBI we performed midline CCI followed by Imatinib treatment and analyses as above. Briefly, mice were gavaged 45 min after TBI and then twice a day for 5 days following injury with either vehicle or 200 mg/kg of Imatinib. T2 and DWI MRI scans were obtained at 24 h and 7 days post-injury. Lesion volumes were calculated from the T2 scans (Figures 4A,B). As expected, the midline CCI resulted in a bilateral injury that was nearly twice as large as the unilateral injury at 24 h after CCI (39.8 ± 5.6 mm^3^ vs. 22.7 ± 2.9 mm^3^) and remained larger 7 days after injury (18.9 ± 3.2 mm^3^ vs. 12.5 ± 2.4 mm^3^) (compare Figure 2B with Figure 4B). Imatinib treatment was also effective at reducing lesion size in the midline CCI model, producing a non-significant trend toward a smaller lesion at 24 h but a significant decrease in lesion volume by 7 days post injury (18.9 ± 3.2 mm^3^ vs. 8.0 ± 3.2 mm^3^).

ADC analysis was also performed with this model; however unlike the unilateral model where the ADC values were calculated only in the injured hemisphere, in the bilateral TBI model the ADC values were calculated for the entire brain. Similar to the lesion size, both cytotoxic and vasogenic edema were increased in the midline CCI model compared to unilateral CCI model (compare Figures 2C–4C). These data also demonstrated that even with the more severe injury, Imatinib treatment was effective at reducing both cytotoxic edema at 24 h after injury (0.559 ± 0.009 mm^2^/s vs. 0.635 ± 0.018 mm^2^/s) and vasogenic edema at day 7 (1.577 ± 0.040 mm^2^/s vs. 1.391 ± 0.028 mm^2^/s) (the normal ADC value is shown by the dashed line in Figure 4C).

**Figure 5:**
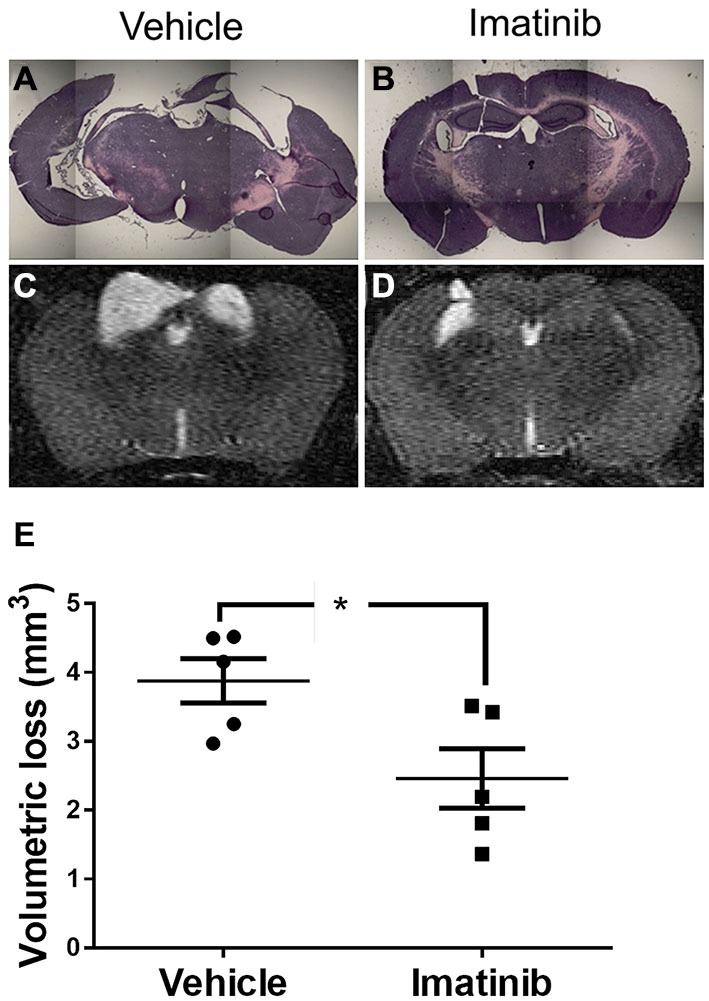
**Imatinib reduces tissue loss after Bi-lateral TBI.** Mice were treated with vehicle or Imatinib (200 mg/kg, twice daily p.o. for 5 days) starting 45 min after bilateral CCI. Twenty one days later, animals underwent MRI T2 scan and then were perfused and fixed in paraformaldehyde and stained with H&E. **(A,B)** Representative photomicrographs of coronal sections near bregma −1.5 (10× objective) from vehicle-treated **(A)** and Imatinib-treated **(B)** mice. **(C,D)** Representative T2-weighted MRI images from vehicle-treated **(C)** and Imatinib-treated mice **(D). (E)** Quantification of volumetric loss of tissue determined from the T2-weighted hyper-intense signal. Data are expressed as mean ± SEM (*n* = 5) and the single asterisk indicates *p* < 0.05.

Mice subjected to midline CCI were also analyzed for long term loss of neuronal tissue due to cavitation 21 days after injury. These data are shown in Figure 5 and indicate that unlike the differences in initial lesion size, that were nearly 2-fold at 24 h after injury (39.8 ± 5.6 mm^3^ vs. 22.7 ± 2.9 mm^3^), the final difference in tissue loss due to cavitation was only ~9% between the two models (3.9 ± 0.3 mm^3^ vs. 3.6 ± 0.3 mm^3^, compare Figures 3E, 5E). Nonetheless, H&E staining of brain sections showed that there was much more hippocampal loss and remodeling in the bilateral TBI model compared to the unilateral model, where the left hippocampus remained largely intact (compare Figures 3A–5A). Similar to the results in lesion size, and edema, Imatinib treatment significantly reduced cavitations in the bilateral TBI model (Figures 5D,E) and appeared to preserve hippocampal structure (Figure 5B). This suggests that even in a severe model of TBI early intervention with Imatinib may have long term benefit in TBI.

### Imatinib Preserves Cognitive Function after Bilateral TBI

**Figure 6:**
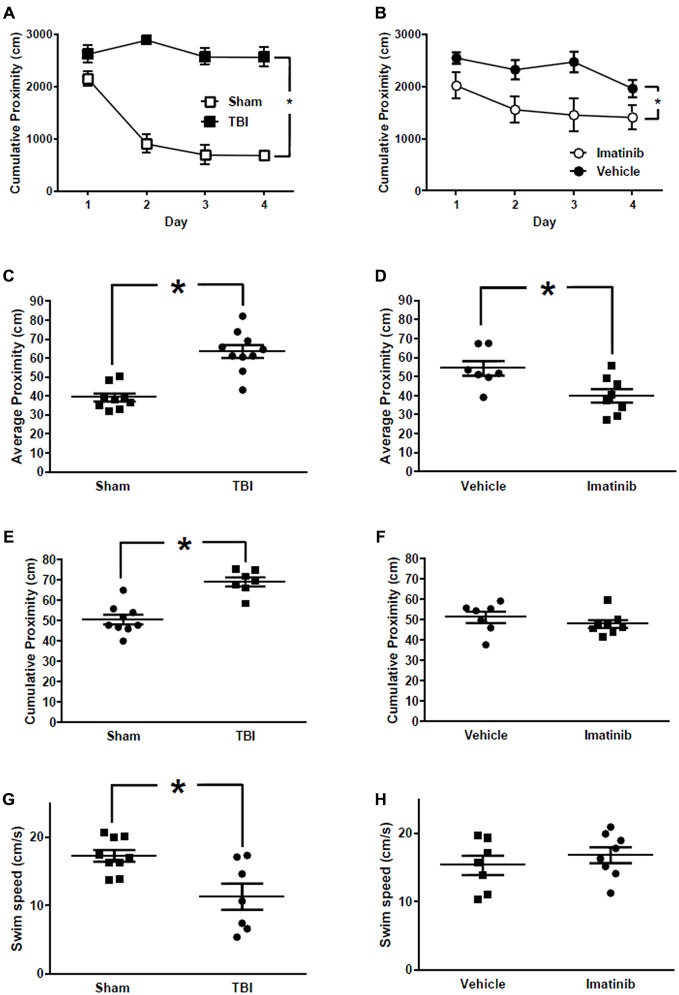
**Imatinib improves neurological outcome in Morris Water Maze (MWM) after TBI.** Imatinib ameliorates TBI associated impairments in spatial learning and memory. **(A)** Cumulative proximity scores are plotted across 4 days of training for TBI (*n* = 7) and Sham (*n* = 9) treated mice. **(B)** Cumulative proximity scores are plotted across 4 days of training for TBI mice treated with vehicle or Imatinib. **(C)** Average proximity during the probe trial which was completed 24 h after the last training trial plotted for Sham and TBI treated mice. **(D)** Average proximity during the probe trial which was completed 24 h after the last training trial is plotted for mice subjected to TBI and then treated with Imatinib (*n* = 8) or Vehicle (*n* = 7). **(E)** Cumulative proximity measured during the visible platform version of the water maze is plotted for Sham and TBI treated mice. Data represent the average of 6 trials. **(F)** Cumulative proximity measured during the visible platform version of the water maze is plotted for mice subjected to TBI and then treated with Imatinib or vehicle. **(G)** Swim speed measured during the probe trial in Sham and TBI treated mice. **(H)** Probe trial swim speed of mice subjected to TBI and then treated with Imatinib or vehicle. Data are presented as mean ± SEM. Single asterisk in **(A,B)** indicates *p* < 0.05 for main effect of treatment evaluated using a two-way repeated measures ANOVA with treatment and training days as factors. Single asterisk in **(C–H)** indicates *p* < 0.05, two tailed unpaired *t*-test.

Cognitive function in mice exposed to midline CCI was assessed by examining spatial learning and memory in the MWM. Data comparing mice with bilateral CCI to Sham treated mice are presented in Figures 6A,C,E,G. Data comparing injured mice treated with Imatinib or vehicle are presented in Figures 6B,D,F,H. All data are presented in terms of proximity which is a measure of “search error” and therefore lower scores indicate a more selective search strategy. Mice in the Sham group exhibited typical performance across the four days of training (Figure 6A) during which time their cumulative proximity scores significantly decreased. Conversely, the performance of mice in the TBI group did not improve over time and was significantly worse compared to the Sham group (*p* < 0.001). To assess long-term memory, a probe trial was conducted 24 h after the last training trial was completed on day 4. Similar to the cumulative proximity, the average proximity measure is a search error measure which is independent of swim speed. The duration of the probe trial is fixed at 60 s; therefore an average measure is made (c.f. cumulative for the variable length training trials). Again, a lower number indicated a more selective search strategy. Injured mice in the TBI group were significantly impaired when compared to the mice in the Sham treated group. In addition, the injured mice in the TBI group performed significantly worse even when the platform was clearly marked (Figure 6E) and exhibited slower swim speeds during the probe trial (Figure 6G). In a separate experiment, mice were exposed to bilateral CCI and then treated with either vehicle or 200 mg/kg of Imatinib by gavage 45 min after CCI and then twice a day for 5 days prior to the start of the water maze. Across the 4 days of training (Figure 6B) injured mice that were treated with Imatinib exhibited significantly better performance when compared to the vehicle treated mice (*p* < 0.01). Similarly, mice treated with Imatinib outperformed vehicle treated mice during the probe trial (Figure 6D). Treatment with Imatinib did not alter performance on the visible platform version of the water maze (Figure 6F) or swim speed (Figure 6H).

### PDGF-CC is Increased in Human CSF after TBI

**Figure 7:**
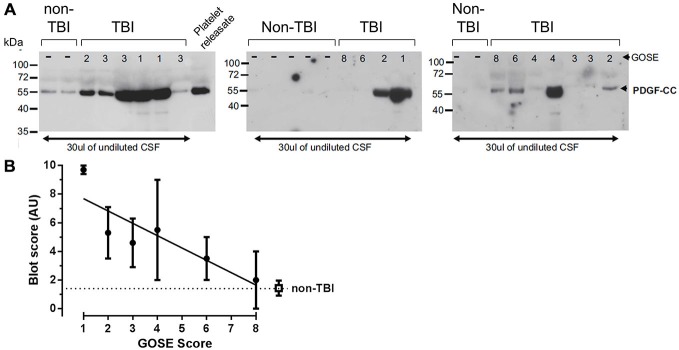
**Analysis of platelet derived growth factor-CC (PDGF-CC) in human TBI cerebrospinal fluid (CSF) samples.** Human CSF Samples obtained from patients within the first 24 h after TBI were immunoblotted for PDGF-CC. **(A)** Immunoblots from 17 TBI patients and nine controls are shown. As a control for PDGF-CC a sample of releasate from human platelets is shown in the last lane of the first blot. The extended glasgow outcome scale (GOSE) score of each patient is shown at the top of each lane (GOSE 1–8) and the position of molecular mass size markers are indicated at the left of each blot. **(B)** The relative amount of PDGF-CC in each sample was estimated from the intensity of the PDGF-CC, assigned a value from 0 to 10 and the average for each GOSE score was plotted against the GOSE score. The filled circles are the TBI samples and the open square represents the non-TBI samples. Each point is the mean ± SEM and the line is a linear regression fit of the TBI samples, a Spearman’s correlation analysis of these data yields a one tailed *p* = 0.0292.

Although our data suggest that the BBB leakage, controlled by PDGF signaling in the NVU, may contribute to TBI progression in animal models, it is not known if PDGF-CC protein levels are altered in human TBI. Therefore, we examined PDGF-CC levels in the CSF of human TBI patients and in control non-TBI patients by immunoblotting. For these studies we obtained CSF from 17 TBI patients and 9 non-TBI patients. The TBI patients had GOSE ranging from 1 to 8, where 1 is the most severe, indicating that the patient died of their injuries, and 8 is the mildest injury and the patients have a good recovery (Lu et al., 2010). Immunoblot analysis of these samples is shown in Figure 7 along with a platelet releasate sample as a positive control for PDGF-CC. Note that the lanes with the strongest PDGF-CC signal are all from TBI patients with the lowest (most severe) GOSE scores. The relative intensity of the PDGF-CC staining in each lane was ranked from 0 to 10 for both the TBI patients and non-TBI patients with 0 being undetectable and 10 representing the most intense PDGF-CC staining, and these data were then plotted against the GOSE score (Figure 7B). This semi-quantitative analysis suggests that there may be a correlation between TBI severity and the presence of PDGF-CC in the CSF. These data are also similar to a positive correlation reported between PDGF-CC levels in plasma and worse outcomes in ischemic stroke patients (Rodríguez-González et al., 2013), and with our previous study showing elevated tPA-inhibitor complexes in the CSF of the most severe TBI patients (Sashindranath et al., 2012).

## Discussion

The loss of BBB integrity is a common feature of severe TBI with nearly 50% of the patients examined in a recent study showing biochemical evidence of BBB disruption (Ho et al., 2014). Damage to the BBB can be immediate due to trauma-induced hemorrhage (Logsdon et al., 2015), or can occur rapidly following TBI in the absence of overt hemorrhage, with significant increases in permeability observed within 3 h of injury (Sashindranath et al., 2012). This early rise in BBB permeability can affect the molecular injury pathways associated with secondary brain injury in TBI, increasing cerebral edema by disrupting parenchymal fluid homeostasis and by increasing neuroinflammation (Shlosberg et al., 2010; Thal and Neuhaus, 2014; Logsdon et al., 2015). A principal focus in the management of TBI is limiting the occurrence of cerebral edema and the attendant increase in ICP (Hemphill and Phan, 2013a), therefore targeting pathways that promote BBB disruption in TBI should be beneficial. Our earlier studies demonstrating that the pathway regulated by the protease tPA, acting through PDGF-CC and the PDGFRα in the NVU, can promote pathologic increases in BBB permeability (Yepes et al., 2000, 2003; Fredriksson et al., 2004, 2005, 2015; Su et al., 2008; Abrams et al., 2012; Adzemovic et al., 2013) led us to test the hypothesis that treatment with Imatinib, an antagonist of the PDGFRα, could reduce BBB permeability after TBI and improve outcomes. Consistent with this hypothesis an earlier study has shown that mice deficient in tPA have reduced BBB disruption 6 h after injury, reduced edema 24 h after injury and smaller lesions 7 days following TBI (Mori et al., 2001). Whereas we have shown that overexpression of tPA results in a significant increase in BBB leakage and lesion volume 3 h after TBI (Sashindranath et al., 2012).

Our data presented here demonstrate for the first time that Imatinib dramatically reduces BBB permeability 24 h after TBI by approximately 80% (Figure 1). This is similar to the extent of BBB protection from Imatinib in a model of spinal cord injury (Abrams et al., 2012) and even better than the approximately 30% reduction in BBB permeability we observed in stroke (Su et al., 2008). Consistent with the view that BBB permeability contributes to increased cerebral edema, Imatinib treatment for 5 days also significantly reduced vasogenic edema in both unilateral and bilateral injury models (Figures 2C, 4C). Imatinib also significantly reduced cytotoxic edema 24 h after injury in the bilateral TBI model but was not significantly different from vehicle in the unilateral model. This was likely because the milder injury in the unilateral model produced less cytotoxic edema at 24 h than did the bilateral injury (unilateral 0.620 ± 0.043 mm^2^/s vs. bilateral 0.559 ± 0.009 mm^2^/s).

The reductions in BBB permeability and cerebral edema induced with Imatinib treatment were also correlated with significant reductions in lesion size during the first week following injury and with tissue preservation at 21 days after CCI (Figures 2–5). Examination of the hematoxylin and eosin stained sections demonstrated that Imatinib treatment was remarkably effective at preserving brain tissue compared to the vehicle treated mice. In fact in the vehicle treated mice the loss of tissue due to vacuole formation or cavitation was so extensive that it was not possible to quantify the magnitude of tissue loss in either the unilateral or midline CCI model (Figures 3, 5). Therefore, T2 MRI scans were utilized to quantify the extent of cavitation after CCI and the effects of 5 days of Imatinib treatment on tissue preservation. The preservation of brain tissue at 21 days following injury is significant for the long term recovery from TBI. Previous studies have shown that neuronal tissue loss several weeks after injury, especially in the hippocampus, is a common pathology in TBI that is associated with memory deficit and cognitive decline (Witgen et al., 2005). Our results demonstrate that Imatinib treatment preserves significantly more tissue compared to vehicle including tissue in the hippocampus. This is particularly apparent when comparing Figures 5A,B. The exact mechanism by which 5 days of Imatinib treatment is able reduce cavitation 21 days after injury is uncertain. However, it is likely that early reduction in BBB leakage together with the reductions in cerebral edema achieved with Imatinib treatment contributed to reductions in tissue loss.

Behavioral impairment in the unilateral CCI model was not consistently observed even in the vehicle treated mice. However, midline cortical impact clearly disrupted performance in the water maze. Compared to Sham treated mice, mice in the TBI group exhibited significantly degraded search strategies as measured by cumulative proximity during training and average proximity during the probe trial. Treatment with Imatinib significantly ameliorated these deficits. Injured mice also exhibited, on average, slower swim speeds during the probe trial and were impaired in the visible platform version of the water maze. Interestingly, injured mice treated with vehicle or Imatinib performed to the same level in the visible platform task and did not differ with regard to their swim speeds. Furthermore, performance in both groups appears to be similar to that observed in the Sham treated mice. This likely reflects the reduction in stress and anxiety produced by the additional handling associated with the daily dosing of the mice in the Imatinib experiment (Fridgeirsdottir et al., 2014). Interpreted this way, these data would suggest that at least some of the performance deficits observed in the TBI mice may be more related to affective state and not due to deficits in motor performance. This interpretation would be consistent with previous work in the field demonstrating that TBI in mice produces deficits in behavioral tests of anxiety-like behaviors (Washington et al., 2012). In light of these observations we conclude that Imatinib improves performance in the water maze by ameliorating TBI associated cognitive impairments and not by altering affective state or motor output.

Together, these data support the view that a dysfunctional BBB and ensuing edema may contribute directly to TBI progression including tissue loss and cognitive impairment, and that treatment with Imatinib reduces these pathologic effects in injured mice. Analysis of human CSF samples from TBI patients suggests that PDGF-CC levels may correlate with the extent of injury. Importantly though, it should be noted that these data are semi-quantitative, and while they suggest that this pathway could be active in human TBI, additional studies will be needed to directly test this hypothesis. Nonetheless, it is interesting to note that in ischemic stroke patients treated with tPA there is a significant positive association between increased plasma PDGF-CC and hemorrhagic transformation (Rodríguez-González et al., 2013), suggesting that in humans PDGF-CC may be linked to cerebrovascular damage.

In conclusion, the data presented here support the hypothesis that the PDGFRα pathway may play a role in the loss of BBB integrity following TBI, and that inhibiting this pathway may improve TBI outcomes. In addition, we demonstrate the potential effectiveness of Imatinib, an existing FDA approved antagonist of the PDGFRα pathway, for the treatment of acute TBI, suggesting the possibility of the relatively rapid translation of Imatinib into a clinical trial in TBI.

## Conflict of Interest Statement

Drs. U. Eriksson, D.A. Lawrence, E.J Su, and L. Fredriksson hold a patent on modulating blood-neural barrier using PDGFR-alpha antagonist. The other authors declare that the research was conducted in the absence of any commercial or financial relationships that could be construed as a potential conflict of interest.
